# Drusen-Like Deposit Choroidopathy in Systemic Lupus Without Glomerulonephritis Treated With a Dexamethasone Implant

**DOI:** 10.7759/cureus.108320

**Published:** 2026-05-05

**Authors:** Nadyr A Damasceno, Soraya A Horowitz, Rodrigo S Pegado, Eduardo F Damasceno

**Affiliations:** 1 Ophthalmology, Universidade Federal do Rio de Janeiro, Rio de Janeiro, BRA; 2 Ophthalmology, Hospital Naval Marcílio Dias, Rio de Janeiro, BRA; 3 Ophthalmology, Instituto Brasileiro de Assistência e Pesquisa (IBAP), Niterói, BRA; 4 Ophthalmology, Universidade Federal Fluminense, Rio de Janeiro, BRA

**Keywords:** choroidopathy, drusenoid-like deposits, increased choroidal thickness, nephritis, systemic lupus erythematosus

## Abstract

We report a case of a 54-year-old woman referred to our clinic with a prior diagnosis of neovascular age-related macular degeneration (nAMD) and who had received three intravitreal aflibercept injections in the left eye (LE). Fundus examination revealed bilateral drusen-like deposits (DLDs) in the posterior pole, along with macular edema in the LE. Optical coherence tomography (OCT) demonstrated subretinal fluid, retinal pigment epithelium detachment (PED) in the LE, and bilateral DLDs and increased choroidal thickness. These findings raised suspicion of an inflammatory condition, prompting laboratory evaluation that supported the diagnosis of systemic lupus erythematosus (SLE). The ocular findings were subsequently reclassified as inflammatory choroiditis associated with DLDs, without evidence of lupus nephritis. Treatment with systemic corticosteroids, mycophenolate mofetil, and dexamethasone intravitreal implant in the LE resulted in progressive clinical improvement.

## Introduction

Systemic lupus erythematosus (SLE) is an autoimmune disorder characterized by the loss of immune tolerance to nuclear antigens, polyclonal autoantibody production, immune complex deposition, and multisystem involvement. It primarily affects women of childbearing age and is characterized by unpredictable flares and remissions. While no cure exists, treatments aim to manage symptoms and reduce damage. The diagnosis is generally established based on the presence of at least four of the eleven criteria defined by the American Rheumatism Association (ARA). These criteria encompass a range of organ manifestations associated with SLE, including (1) malar rash, (2) discoid rash, (3) skin photosensitivity, (4) oral ulcers, (5) nonerosive arthritis, (6) serositis, (7) renal involvement, (8) neurological disorder, (9) hematologic disorder, (10) immunologic disorder, and (11) positive antinuclear antibodies [1,2].

Ocular involvement affects 2-30% of patients with SLE, ranging from dry eye syndrome to vision-threatening vasculitis (lupus retinopathy), despite not being included in the American College of Rheumatology (ACR) criteria. Retinal vasculature is frequently involved, with fundus examination revealing findings, such as cotton-wool spots, hemorrhages, perivascular white sheathing, and edema [3,4,5,6]. By contrast, lupus choroidopathy is rare, with approximately 60 cases reported in the literature, and is characterized by bilateral serous or exudative retinal detachment and subretinal deposits [7]. Drusen are yellowish deposits observed on fundus examination in patients with age-related macular degeneration (AMD). Drusen in AMD result from the retinal pigment epithelium's (RPE) inability to remove cell debris and extracellular matrix, leading to complement activation, as well as involvement of reactive oxidation, apoptosis, and angiogenesis [8,9]. It is a complex aging system. By contrast, drusen-like deposits (DLDs) associated with SLE are more numerous and larger and are preferably located in the temporal retina on a fundus exam. They are more frequent in patients with long-standing SLE and class IV lupus nephritis [10]. Lupus choroidopathy with DLDs is thought to be related to the deposition of immune complexes, inflammatory cells, immunoglobulins, and complement in the choroidal vessels, resulting in choroidal hyperpermeability and rupture of the external retinal barrier, which is similar to what occurs in the kidney [11,12].

Recognition of lupus choroidopathy with DLDs is critical, as these findings may indicate active SLE with severe systemic involvement, particularly lupus nephritis, and may mimic AMD, posing a diagnostic challenge.

## Case presentation

**Figure 1:**
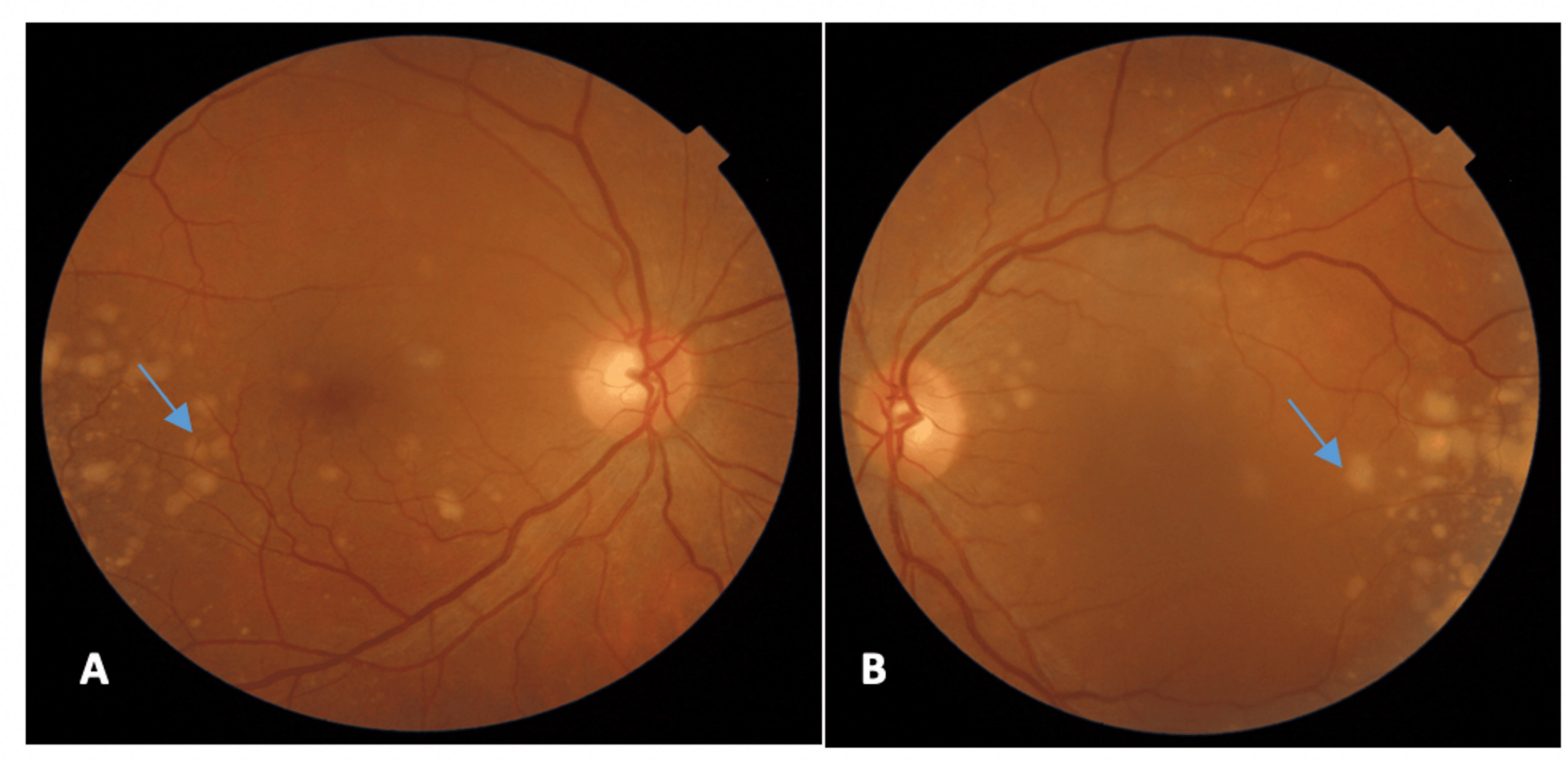
A and B: Color fundus photography demonstrating DLDs distributed throughout the posterior pole in both eyes (thin blue arrows). A refers to the right eye, and B refers to the left eye.

A 54-year-old woman was referred to our service one month after receiving three intravitreal injections of aflibercept in the left eye (LE) for a prior diagnosis of neovascular age-related macular degeneration (nAMD). On examination, best corrected visual acuity (BCVA) was 20/30 in the right eye (RE) and 20/200 in the LE, measured using the Snellen chart. Slit-lamp biomicroscopy of the anterior chamber was unremarkable in both eyes, and intraocular pressure was within normal limits bilaterally. Fundus examination revealed multiple bilateral yellowish drusenoid-like deposits (DLDs) distributed throughout the posterior pole, predominantly in the temporal retina, and larger and more diffusely distributed than typically observed in AMD (Figures 1A, 1B). Moreover, the LE showed macular edema with loss of the foveal reflex. 

Thin blue arrows indicate DLDs, the thick blue arrows indicate PED, the quad blue arrows indicate subretinal fluid, the blue star indicates large PED, the blue sun arrows indicate the absence of choroidal neovascularization, and the right brace indicates increased choroidal thickness. 

**Figure 2:**
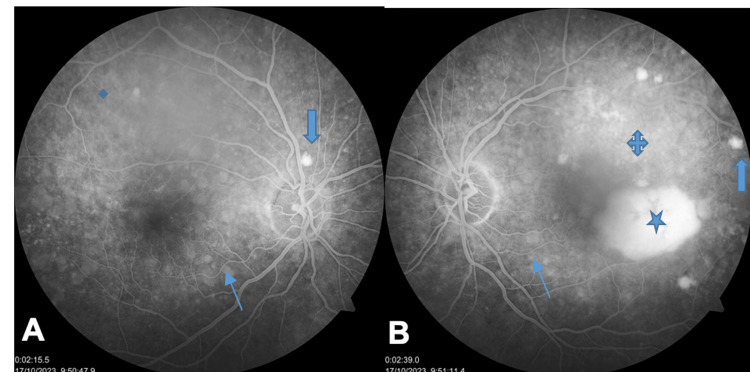
A and B: Fluorescein angiography (FA) showing diffuse window defect hyperfluorescence corresponding to drusen-like deposits (thin blue arrows), focal areas of leakage (quad arrows), and small pigment epithelial detachments (PEDs) (thick blue arrows); a large macular PED is noted in the left eye (LE) (blue star). A refers to the right eye, and B refers to the left eye.

On fluorescein angiography (FA), there were diffuse window defect hyperfluorescent lesions, some areas of leakage in both eyes, and a large RPE detachment in the macula in the LE (Figures 2A, 2B). 

**Figure 3:**
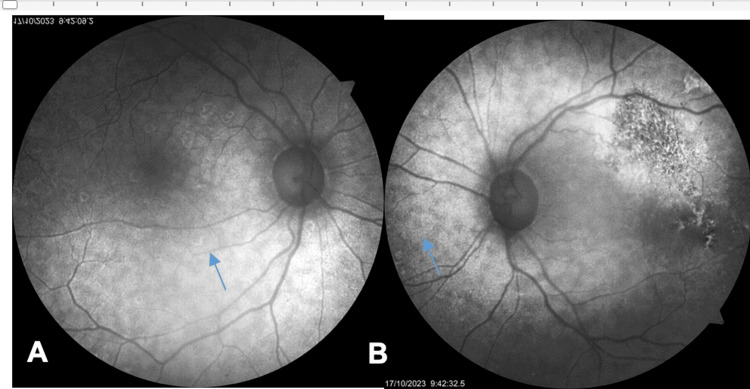
A and B: Fundus autofluorescence (FAF) showing hyperautofluorescent lesions corresponding to drusen-like deposits (DLDs) at the level of the retinal pigment epithelium (thin blue arrows). A refers to the right eye, and B refers to the left eye.

Fundus autofluorescence (FAF) revealed numerous hyperautofluorescent lesions scattered in the posterior pole of both eyes (Figures 3A, 3B).

**Figure 4:**
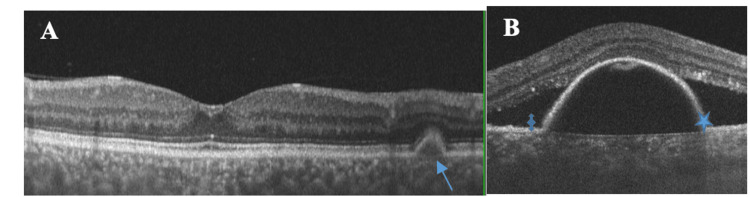
A: Optical coherence tomography (OCT) of the right eye (RE) showing drusen-like deposits (DLDs) at the level of the retinal pigment epithelium (thin blue arrow). B: OCT of the left eye (LE) demonstrating subretinal fluid (quad blue arrow) and large pigment epithelial detachment (blue star), corresponding to the large pigment epithelium detachment (PED) observed on fluorescein angiography.

**Figure 5:**
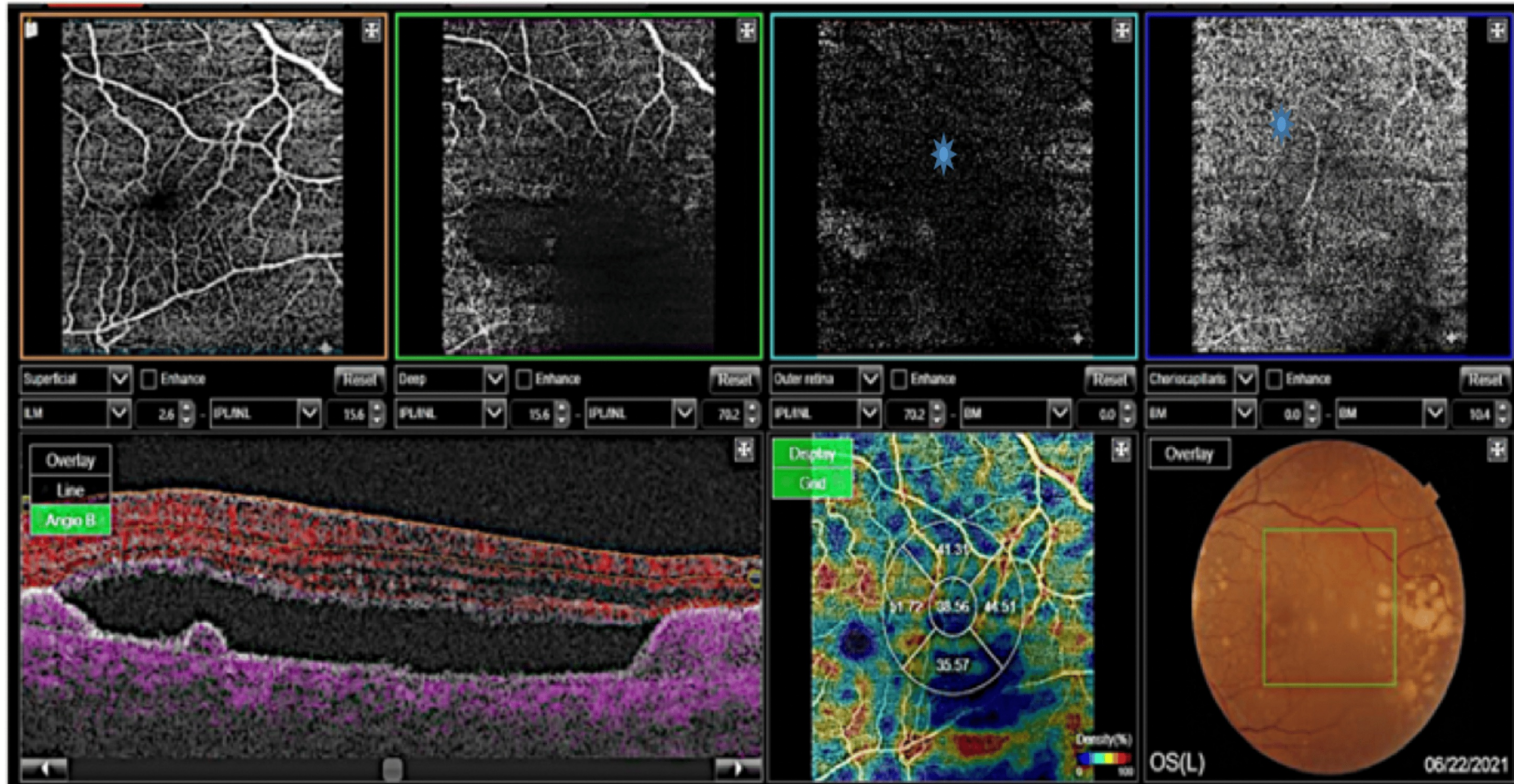
Optical coherence tomography angiography (OCT-A) shows the absence of choroidal neovascularization (blue sun arrows).

Optical coherence tomography (OCT) demonstrated subretinal fluid and retinal PED in the LE, along with bilateral DLDs and increased choroidal thickness (450 µm in the RE and 480 µm in the LE) (Figures 4A, 4B). Optical coherence tomography angiography (OCT-A) did not demonstrate the presence of choroidal neovascularization in the LE (Figure 5).

This patient also reported arthralgia. Laboratory evaluation revealed a strongly positive ANA (+++,1:9,000), with a homogeneous nuclear pattern (1:320), along with positive anti-double-stranded DNA antibodies (anti-dsDNA) (1:60), supporting the diagnosis of active SLE. Fasting blood glucose was 80 mg/dL, red blood cell count was 4.0 million/mm³, and white blood cell count was 6,000/mm³. Renal parameters were within normal limits, including a spot urine protein-to-creatinine ratio of 0.14 (protein 6.3 mg/dL, creatinine 45.96 mg/dL), serum creatinine of 0.4 mg/dL, urea of 36 mg/dL, complement C3 100 mg/dL, and C4 30 mg/dL, indicating no evidence of active lupus nephritis.

**Figure 6:**
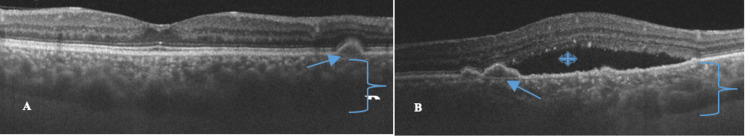
A: Optical coherence tomography (OCT) of the right eye (RE) showing increased choroidal thickness (right brace) and drusen-like deposits (thin blue arrow). B: OCT of the left eye (LE) demonstrating increased choroidal thickness (right brace), drusen-like deposits (thin blue arrow), and subretinal fluid (quad blue arrow).

Neovascular age-related macular degeneration was considered unlikely, given the lack of functional response to prior anti-VEGF therapy, absence of choroidal neovascularization on imaging, and the presence of marked choroidal thickening, which is atypical for nAMD. Central serous chorioretinopathy (CSC) was also considered due to the presence of subretinal fluid and increased choroidal thickness; however, the presence of extensive bilateral DLDs, particularly in the temporal retina, and associated large PED is not characteristic of this condition. In the context of systemic findings and serological evidence consistent with SLE, these features supported the diagnosis of lupus choroidopathy associated with DLDs (Figures 6A, 6B).

The patient was started on systemic treatment with prednisolone (60 mg/day, gradually tapered), mycophenolate mofetil (3 g/day), and a dexamethasone intravitreal implant in the LE.

**Figure 7:**
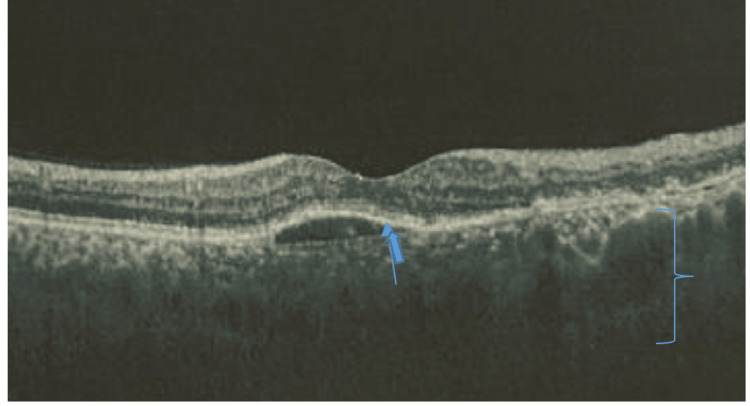
Optical coherence tomography (OCT) of the left eye (LE) showing residual pigment epithelium detachment (PED) (thick blue arrow) and persistent increased choroidal thickness (right brace).

At the 10-month follow‑up, the patient was maintained on oral prednisolone 10 mg/day and mycophenolate mofetil 2 g/day. BCVA was maintained at 20/30 in the RE and improved to 20/70 in the LE. OCT imaging demonstrated resolution of subretinal fluid and a significant reduction in PED, with persistent bilateral increased choroidal thickness (Figure 7). Table 1 summarizes the main clinical, imaging, and laboratory findings of this case report.

**Table 1:** Summary of the main findings BCVA: best corrected visual acuity, RE: right eye, LE: left eye, DLD: drusen-like deposit, FA: fluorescein angiography, PED: pigment epithelium detachment, FAF: fundus autofluorescence, OCT: optical coherence tomography, SRF: subretinal fluid, OCT-A: optical coherence tomography angiography, CNV: choroidal neovascularization, ANA: antinuclear antibody, dsDNA: double-stranded DNA, dex: dexamethasone, VA: visual acuity

Category	Findings
Clinical	BCVA 20/30 (RE), 20/200 (LE); bilateral DLDs; macular edema (LE)
Imaging	FA: leakage + PED; FAF: hyperAF lesions; OCT: SRF + PED + thick choroid; OCT-A: no CNV
Systemic	Arthralgia; ANA+++; anti-dsDNA+
Diagnosis	Lupus choroidopathy with DLDs
Treatment	Prednisolone + mycophenolate + Dex implant
Outcome	VA improved; SRF resolved; PED reduced

## Discussion

SLE is a chronic autoimmune disease that predominantly affects women and may involve multiple organ systems, including the eyes. Ocular manifestations range from subclinical findings to vision-threatening conditions, such as retinopathy and choroidopathy. Fundus abnormalities may reflect systemic disease activity and can be associated with the involvement of vital organs, including the kidneys and central nervous system, or may precede systemic manifestations. The disease is characterized by loss of immune tolerance to nuclear antigens, immune complex deposition, and production of autoantibodies [7,13,14].

According to the 2019 European Alliance of Associations for Rheumatology (EULAR)/American College of Rheumatology (ACR) classification criteria, SLE requires a positive ANA as an entry criterion, followed by a cumulative score of ≥10 points across clinical and immunological domains [15]. In this case, the patient met the classification criteria (12 points), with arthritis and positive ANA and anti-dsDNA antibodies. Clinically, she presented with subclinical choroiditis in the RE and clinically evident choroiditis in the LE, bilateral diffuse DLDs, and increased choroidal thickness.

Drusenoid PED, a biomarker associated with progression to atrophy in AMD, is defined by the coalescence of soft drusen into a lesion measuring at least 350 µm in diameter. On OCT, drusenoid PED typically shows moderate to high internal reflectivity [16,17]. Although subretinal fluid, PED, and bilateral drusenoid elevations may resemble AMD, increased choroidal thickness in SLE-associated choroidopathy favors an inflammatory rather than degenerative etiology [17].

CSC is an important differential diagnosis, typically affecting men and presenting with serous retinal detachment, PED, and increased choroidal thickness [18,19]. Vogt-Koyanagi-Harada (VKH) disease should also be considered, as it presents with bilateral diffuse choroiditis and exudative retinal detachment; however, unlike SLE, it is typically associated with systemic manifestations and granulomatous panuveitis. Additional differential diagnoses include hypertensive choroidopathy and posterior scleritis [20].

Optical coherence tomography is a key noninvasive imaging modality for evaluating retinal and choroidal changes. In SLE, PED is usually associated with lupus choroidopathy and results from the disruption of the blood-retinal barrier, leading to fluid accumulation and immune complex deposition between the RPE and Bruch’s membrane. DLDs are thought to represent accumulations of immune complexes and complement components, potentially reflecting active inflammatory activity. Although SLE choroidopathy is more commonly associated with severe systemic disease, particularly lupus nephritis, it may also occur in patients without renal involvement. DLDs have been more frequently described in patients with lupus nephritis and are relatively uncommon in those without renal disease [11].

The absence of indocyanine green angiography (ICGA) may be considered a limitation of this case report, as it could have provided additional characterization of choroidal involvement.

## Conclusions

Drusenoid pigment epithelial detachment (PED) is a recognized biomarker of AMD; however, it is important to distinguish it from DLDs, which may occur as a manifestation of SLE. In this case, increased choroidal thickness and multimodal imaging findings supported an inflammatory etiology consistent with SLE-associated choroidopathy. Moreover, this case report highlights the potential misdiagnosis as nAMD, which may lead to inappropriate therapy if inflammatory features are not recognized.

These findings emphasize the importance of considering inflammatory causes in atypical presentations of drusen-like lesions, particularly when clinical and systemic features suggest autoimmune diseases. However, given the nature of a single case report, these observations should be interpreted with caution and considered hypothesis-generating. Further studies are needed to better define the diagnostic and therapeutic implications of these findings.
